# Differential modulation of repetitive firing and synchronous network activity in neocortical interneurons by inhibition of A-type K^+^ channels and I_h_

**DOI:** 10.3389/fncel.2015.00089

**Published:** 2015-03-18

**Authors:** Sidney B. Williams, John J. Hablitz

**Affiliations:** Department of Neurobiology, Civitan International Research Center and Evelyn F. McKnight Brain Institute, University of Alabama at Birmingham, Birmingham, ALUSA

**Keywords:** HCN channels, A-type K^+^ channels, 4-AP, synchronization, I_**h**_, neocortex, GABAergic interneurons, Martinotti cells, basket cells

## Abstract

GABAergic interneurons provide the main source of inhibition in the neocortex and are important in regulating neocortical network activity. In the presence 4-aminopyridine (4-AP), CNQX, and D-APV, large amplitude GABA_A_-receptor mediated depolarizing responses were observed in the neocortex. GABAergic networks are comprised of several types of interneurons, each with its own protein expression pattern, firing properties, and inhibitory role in network activity. Voltage-gated ion channels, especially A-type K^+^ channels, differentially regulate passive membrane properties, action potential (AP) waveform, and repetitive firing properties in interneurons depending on their composition and localization. HCN channels are known modulators of pyramidal cell intrinsic excitability and excitatory network activity. Little information is available regarding how HCN channels functionally modulate excitability of individual interneurons and inhibitory networks. In this study, we examined the effect of 4-AP on intrinsic excitability of fast-spiking basket cells (FS-BCs) and Martinotti cells (MCs). 4-AP increased the duration of APs in both FS-BCs and MCs. The repetitive firing properties of MCs were differentially affected compared to FS-BCs. We also examined the effect of I_h_ inhibition on synchronous GABAergic depolarizations and synaptic integration of depolarizing IPSPs. ZD 7288 enhanced the amplitude and area of evoked GABAergic responses in both cell types. Similarly, the frequency and area of spontaneous GABAergic depolarizations in both FS-BCs and MCs were increased in presence of ZD 7288. Synaptic integration of IPSPs in MCs was significantly enhanced, but remained unaltered in FS-BCs. These results indicate that 4-AP differentially alters the firing properties of interneurons, suggesting MCs and FS-BCs may have unique roles in GABAergic network synchronization. Enhancement of GABAergic network synchronization by ZD 7288 suggests that HCN channels attenuate inhibitory network activity.

## Introduction

GABAergic interneurons are the main source of inhibition in the neocortex and regulate the output of neocortical networks. GABA released from interneurons acts on a variety of receptors located both pre- and post-synaptically (Martin and Olsen, 2000; Farrant and Kaila, 2007). Activation of GABA_A_ receptors produces gating of Cl^-^ permeable channels resulting in phasic inhibition via membrane hyperpolarization (Avoli and de Curtis, 2011). During the early postnatal period, GABA_A_ receptor mediated responses can be depolarizing due to a lack of KCC2, a K^+^-Cl^-^ co-transporter that extrudes Cl^-^ (Ben-Ari et al., 1989; Rivera et al., 1999). These depolarizing responses are involved in network synchronization during development (Khazipov et al., 1997; Allene et al., 2008). In response to iontophoretic application of GABA, depolarizing GABA_A_-mediated responses can also be seen in mature animals (Andersen et al., 1980; Alger and Nicoll, 1982; Weiss and Hablitz, 1984). Application of the A-type K^+^ channel blocker 4-aminopyridine (4-AP) results in generation of a GABAergic, long-lasting depolarization (termed giant depolarizing potentials; Avoli and Perreault, 1987; Avoli et al., 1988). These responses persist when excitatory glutamatergic transmission is blocked with CNQX and D-APV (Aram et al., 1991; Michelson and Wong, 1991; Avoli et al., 1994; Benardo, 1997). These depolarizing GABA responses, which propagate across the neocortex (DeFazio and Hablitz, 2005), are assumed to result from synchronous firing of inhibitory interneurons. The present study examines activity in specific classes of neocortical interneurons during such depolarizing events.

GABAergic interneurons are involved in processes such as modulation of synaptic integration (Pouille and Scanziani, 2001; Klausberger and Somogyi, 2008), control of spike timing (Wehr and Zador, 2003), and synchronization of network activity (McBain and Fisahn, 2001). Subpopulations of GABAergic interneurons can be identified based on cell morphology, intrinsic excitability, and inherent protein expression patterns. Each GABAergic interneuron subtype has a unique synaptic target. The largest class of GABAergic interneurons in the neocortex are the parvalbumin (PV)-expressing cells, which constitute roughly 40% of the interneurons in the neocortex (Rudy et al., 2011). A subclass of PV-expressing cells, the fast-spiking basket cells (FS-BCs), have a multipolar morphology, vary in size, and target the proximal dendrite and soma of pyramidal cells (Kawaguchi and Kubota, 1997). These cells exhibit low input resistance, high frequency repetitive firing and no accommodation (Kawaguchi and Kubota, 1997). They have been shown to exhibit powerful feed-forward inhibition (Pouille and Scanziani, 2001) and initiate oscillatory activity (Traub et al., 2005). Importantly, FS-BCs also contribute to the control of the excitation/inhibition balance necessary to maintain the functional integrity of the cortical network (Haider and McCormick, 2009), induce gamma rhythm activity (Cardin et al., 2009), and entrain excitatory neurons (Fries et al., 2001; Kawaguchi, 2001; Hasenstaub et al., 2005). In contrast to FS-BCs, Martinotti cells (MCs) express the peptide somatostatin (SOM) and synaptically target the apical and basal dendrites of pyramidal neurons. MCs display vast arborized axons that densely innervate layer I across multiple columns. These cells demonstrate burst firing in response to depolarizing steps and show accommodation (Kawaguchi and Kubota, 1997). Excitatory inputs from pyramidal cells onto MCs are strongly facilitating and drive feedback inhibition (Silberberg and Markram, 2007).

4-aminopyridine sensitive, A-type K^+^ channels encompass a subset of voltage-gated K^+^ channels comprised of the Kv1, Kv3, and Kv4 subunit subfamilies (Jerng et al., 2004). Differential expression of these channels gives rise to the different characteristic repetitive firing patterns among interneurons (Martina et al., 1998; Serodio and Rudy, 1998; Wang et al., 1998; Chow et al., 1999; Erisir et al., 1999; Lau et al., 2000; Lien et al., 2002). *In situ* hybridization and immunofluorescent labeling demonstrate Kv3.1 and Kv3.2 transcripts and proteins co-localize with PV-positive interneurons (Weiser et al., 1994; Sekirnjak et al., 1997; Chow et al., 1999). Furthermore, pharmacological inhibition and genetic disruption of presynaptic Kv1 and somatodendritic Kv3 channels impairs fast-spiking firing patterns in interneurons (Martina et al., 1998; Erisir et al., 1999; Lau et al., 2000; Goldberg et al., 2008). Alternatively, SOM positive interneurons have been shown to contain a significant higher density of somatodendritic Kv4 channels and the associated K^+^ current, contributing to their characteristic firing pattern (Serodio and Rudy, 1998; Lien et al., 2002; Lai and Jan, 2006; Bourdeau et al., 2007). Kv3.2 channels are also highly expressed in non-fast-spiking SOM positive interneurons in the neocortex, where they may play a different role in repetitive firing (Weiser et al., 1994; Chow et al., 1999). Consistent with their role in regulating intrinsic excitability, the genetic loss or pharmacological blockade of A-type K^+^ channels is epileptogenic (Smart et al., 1998; Avoli et al., 2001; Bagetta et al., 2004; Monaghan et al., 2008). It remains unclear how the inhibition of A-type K^+^ channels induces interneuron synchronization.

Cortical network excitability can be modulated by hyperpolarization-activated cyclic nucleotide-gated (HCN) channels, and their associated I_h_ current. In excitatory pyramidal cells, the I_h_ current contributes to the cell’s intrinsic excitability by depolarizing the membrane, increasing the membrane conductance, and decreasing dendritic excitability (Magee, 1998; Williams and Stuart, 2000; Berger et al., 2001; Robinson and Siegelbaum, 2003). During synaptic activation, I_h_ normalizes the decay time of distal excitatory postsynaptic potentials (EPSPs; Williams and Stuart, 2000) and decreases temporal summation (Berger et al., 2001). It also functions to constrain excitatory network activity (Albertson et al., 2013). Furthermore, loss of HCN channels has been reported in experimental epilepsy models (Jung et al., 2007; Powell et al., 2008; Shin et al., 2008; Albertson et al., 2011). Neocortical GABAergic interneurons do not typically stain with HCN channel antibodies (Lorincz et al., 2002), but do display varying amounts of I_h_. FS-BCs demonstrate small or absent “sag” responses upon hyperpolarization (Okaty et al., 2009; Albertson et al., 2013). In contrast, MCs display a prominent “sag” response to hyperpolarizing current pulses and a “rebound” response to repolarization, characteristic of I_h_ (Lupica et al., 2001; Wang et al., 2004; Ma et al., 2006). The role of HCN channels in modulating GABAergic interneuron excitability and inhibitory network activity is not well established.

In the present study, we examined the influence of A-type K^+^ channels on AP and repetitive firing properties of L2/3 FS-BCs and MCs in the 4-AP model of interneuron network synchronization. We further investigated the role of HCN channel inhibition in modulating 4-AP induced GABAergic network synchronization. We found that 4-AP differentially alters the repetitive firing properties of FS-BCs and MCs. We also found that I_h_ inhibition enhances the magnitude of evoked and spontaneous depolarizing GABAergic potentials as well as the frequency of spontaneous depolarizing GABAergic potentials in L2/3 neocortical interneurons. These results indicate that interneuron excitability is both up- and down-regulated by voltage-gated ion channels.

## Materials and Methods

### Ethics Statement

All experiments were performed in accordance with the National Institutes of Health Guide for the Care and Use of Laboratory Animals using protocols approved by the University of Alabama at Birmingham Institutional Animal Care and Use Committee.

### Slice Preparation

To prepare acute neocortical slices, vesicular GABA transporter (VGAT)-Venus-expressing Wistar rats (Uematsu et al., 2008) from PND 20 to 36 were anesthetized with isoflurane and rapidly decapitated. Brains were removed and immediately placed in ice-cold oxygenated (95% O_2_/5% CO_2_, pH 7.4) cutting solution consisting of (in mM): 135 *N*-Methyl-D-glucamine, 1.5 KCl, 23 NaHCO_3_, 0.4 ascorbic acid, 25 D-glucose, 1.5 KH_2_PO_4_, 1.25 CaCl_2_, and 8.75 MgCl_2_ (Tanaka et al., 2008). Using a Microm HM 650 vibratome (Microm, Walldorf, Germany), coronal brain slices (300 μm thick) of somatosensory cortex were made. After slicing was completed, neocortical slices were placed in a holding chamber in a water bath at 37°C for 45–60 min in saline consisting of (in mM) 125 NaCl, 3.5 KCl, 10 D-glucose, 26 NaHCO_3_, 1.25 NaH_2_PO_4_, 2.5 CaCl_2_, 1.3 MgCl_2_. Slices were subsequently kept at room temperature until recording.

### Whole Cell Recording

Slices were visualized using a Zeiss AxioExaminer D1 (Carl Zeiss Inc., Thornwood, NY, USA) microscope, equipped with Dodt contrast optics, a 40X-water immersion lens and infrared illumination. Individual slices were held in a submerged recording chamber continuously perfused with oxygenated saline (3 ml/min at 32°C). Whole-cell access was obtained using glass patch electrodes with an open tip resistance of 3–5 MΩ. Pipettes were filled with an intracellular solution consisting of (in mM): 125 K-gluconate, 10 KCl, 10 HEPES, 10 creatine phosphate, 2 Mg-ATP, 0.2 Na-GTP, 0.5 EGTA, with an adjusted pH of 7.3 and osmolarity of 290 mOsm. In most experiments, biocytin (0.5%; Sigma, St. Louis, MO, USA) was added to the intracellular solution for *post hoc* morphological analysis.

### Data Acquisition and Analysis

Whole-cell recordings were obtained using an ELC-03XS npi bridge balance amplifier (npi Electronic GmbH, Tamm, Germany) with Clampex 8.2 software via a Digidata 1322A interface (Molecular Devices, Union City, CA, USA), filtered at 2 kHz and digitized at 10 kHz. Analysis of all recordings was performed using Clampfit 9.0 software (Molecular Devices). Interneurons were physiologically identified by the response to a series of 800 ms hyperpolarizing and depolarizing current pulses ranging from -200 – 350 pA. Resting membrane potential (RMP) was monitored and maintained throughout the recording process. Input resistance was measured in current clamp with 800 ms, 25 pA hyperpolarizing current steps. Initial and final firing frequencies were calculated from the time interval between the first and last two APs, respectively, in an 800 ms depolarizing current pulse. Accommodation ratio was defined as the initial firing frequency/final firing frequency in response to a depolarizing current pulse. The amplitude of the after-hyperpolariztion (AHP) was measured form the AP threshold the the peak of the AHP. The slow after-depolarization (sADP) following an 800 ms current pulse was measured from the RMP before the current pulse to the peak of the sADP. Synaptic responses were evoked using a bipolar nichrome electrode positioned 100–200 μm adjacent to the recording electrode. Evoked depolarizing GABAergic potentials were triggered using a single 50–320 μA current pulse of 100 μs duration. A train of 5 stimuli at 25 Hz was used to study synaptic integration. Amplitude of evoked depolarizing GABAergic potentials was measured from RMP at the time of stimulation to the peak of depolarization. Evoked and spontaneous responses area was measured from the time of stimulation to the point at which the membrane potential returned to baseline.

### Drugs and Drug Application

Drugs were obtained from the following sources: 4-AP, Sigma, St. Louis, MO, USA; 6-cyano-7-nitroquinoxaline-2,3-dione (CNQX), Abcam, Cambridge, MA, USA; D-(-)-2-Amino-5-phosphonopentanoic acid (D-APV) and 4-Ethylphenylamino-1,2-dimethyl-6-methylaminopyrimidinium chloride (ZD 7288; Tocris, Ellisville, MO, USA).

To induce synchronous GABA-mediated events, slices were incubated in 100 μM 4-AP for at least 1 h prior to recording. After control recordings were obtained in the presence of 10 μM CNQX and 20 μM D-APV, 20 μM ZD 7288 was applied to inhibit HCN channels. All drugs were bath applied and each neuron served as its own control.

### Statistics

All statistical analysis was performed using GraphPad Prism 4 (LaJolla, CA, USA). Data are expressed as mean ± SEM. Sample size (n) is the number of cells used for each experiment, with a maximum of three cells per animal. Statistical comparisons of responses before and during drug application was performed using a one- or two-tailed Student’s *t*-test, for which *p* < 0.05 was considered significant. For analysis of IPSP summation before and after I_h_ inhibition, a two-way ANOVA was used.

## Results

### Identification of FS-BC and MCs in Rat Neocortex

Neocortical GABAergic interneurons represent approximately 25% or less of the total cortical neuron population (Fairen et al., 1984; Peters and Jones, 1984) and are highly heterogeneous (Ascoli et al., 2008). FS-BCs and MCs are two major classes of inhibitory GABAergic interneurons in rat neocortex (Rudy et al., 2011). FS-BCs innervate somatic and perisomatic regions of pyramidal cells, forming dense and unspecific connections (Packer and Yuste, 2011). In contrast, MCs target apical, basal, and distal tuft dendrites of pyramidal cells and contact somas of L1 neurons (Wang et al., 2004; McGarry et al., 2010). In order to facilitate visual identification of specific cell types in L2/3, recordings were obtained in transgenic rats co-expressing the yellow fluorescent protein, Venus, with the vesicular GABA transporter (Uematsu et al., 2008).

**FIGURE 1:**
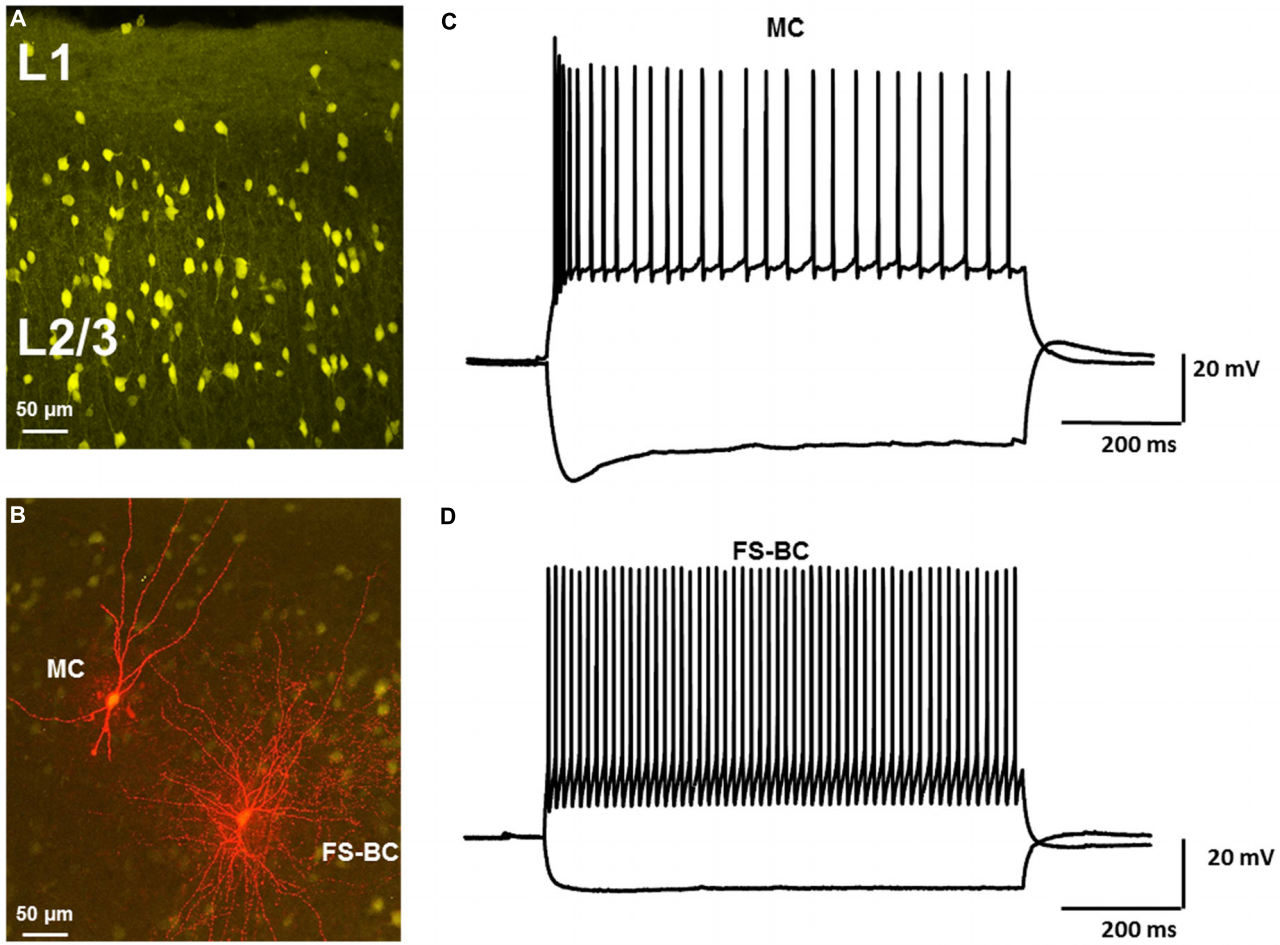
**Identification of neocortical GABAergic interneurons in VGAT-Venus transgenic rats. (A)** Confocal image of Venus-expressing interneurons in upper layers of rat neocortex. Morphologically distinct subtypes of interneurons can be identified. **(B)** Confocal image of Venus-expressing interneurons (yellow) and biocytin labeled interneurons labeled (red). Neurons were labeled with biocytin during whole cell recording. Distinct morphological differences were observed. Leftmost neuron had characteristics of a Martinotti cell (MC) whereas rightmost neuron was classified as a fast spiking basket cell (FS-BC). **(C)** Response of MC to depolarizing and hyperpolarizing current pulses demonstrate typical repetitive firing properties and prominent sag responses. **(D)** Representative response of FS-BC to depolarizing and hyperpolarizing current pulses. High frequency repetitive firing, prominent after hyperpolarizations and lack of a pronounced sag response were typical of fast spiking basket cells.

The laminar distribution of Venus positive cells is shown in **Figure 1A**. Recordings were obtained from fluorescent cells in L2/3. It was possible to morphologically discriminate FS-BCs and MCs prior to recording. FS-BSs were identified on basis of depth below the pial surface and presence of a round soma. Neurons with oval shaped somas and bi-tufted appearance were classified as MCs. Biocytin was included in the patch electrode to allow for *post hoc* confirmation of cell identification. Examples of a Venus-positive GABAergic FS-BC and a MC labeled with biocytin are shown in **Figure 1B**. Cells were further identified by their firing properties and response to strong hyperpolarizing current pulses. Typical MC responses are shown in **Figure 1C**. In the present series, most MCs showed initial burst responses followed by accommodation. A marked “sag” upon hyperpolarization, indicative of I_h_ activation, was seen upon membrane hyperpolarization. In contrast, FS-BCs (**Figure 1D**) displayed high frequency, non-accommodating firing and lacked significant sag responses. The combination of anatomical and electrophysiological characteristics allowed for clear identification of these two classes of cells and allowed us to address the question of differential modulation by 4-AP and inhibitors of I_h_ channels.

### Alterations in the Intrinsic Excitability of GABAergic Interneurons Induced by 4-AP

#### Fast-Spiking Basket Cells

**FIGURE 2:**
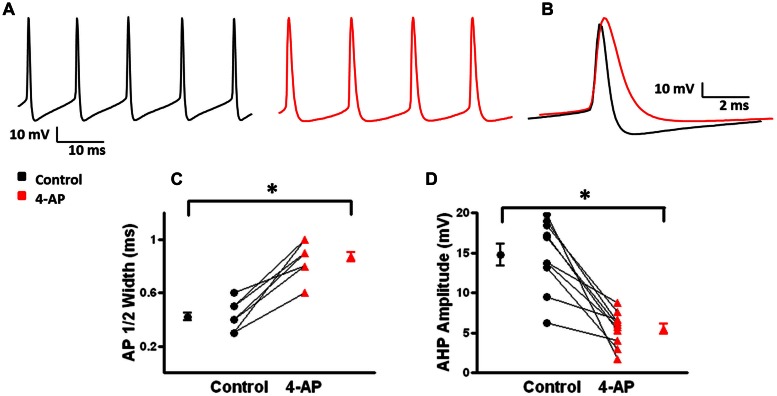
**Effects of 4-AP on action potential (AP) properties of FS-BCs. (A)** Representative firing from a L2/3 FS-BC during a 400 pA depolarizing current pulse before (black) and after (red) bath application of 100 uM 4-AP. 4-AP induced spike widening and decreases in fAHP amplitude. **(B)** Superimposition of APs from A before and after 4-AP demonstrates significantly increased AP duration and decreased AHP amplitude. **(C)** Summary plot showing that bath application of 4-AP significantly increases AP half width of FS-BCs (*n* = 10). **(D)** Summary plot illustrating that 4-AP significantly decreases AHP amplitudes in FS-BCs (*n* = 10). ^∗^*p* < 0.05.

Previous studies of L1 fast-spiking interneurons have shown that low concentrations (50 μM) of 4-AP broadened APs and induced burst firing (Zhou and Hablitz, 1996). In the present study, under control conditions, L2/3 FS-BCs had an average RMP of -68.7 ± 0.7 mV and input resistance of 97.3 ± 18.1 MΩ (*n* = 10). APs were evoked by somatic injections of depolarizing current pulses before and after bath application of 100 μM 4-AP, 10 μM CNQX, and 20 μM D-APV. Under control conditions, suprathreshold current pulses elicited repetitive firing in L2/3 FS-BCs (**Figure 2A**, black). APs under control and 4-AP conditions are shown superimposed in **Figure 2B**. APs had a half-width of 0.42 ± 0.03 ms (*n* = 10) and were followed by a characteristic fast afterhyperpolarization (fAHP) with an amplitude of 14.8 ± 1.4 mV (*n* = 10; **Figures 2A,D**). In the presence of 4-AP, the shape of the AP was distinctly different with a significant increase in the AP half-width (0.9 ± 0.04 ms; *t*-test *p* < 0.05, *n* = 10). The fAHP was blocked and a slower AHP with a significantly lower amplitude was observed (5.5 ± 0.1 mV, *t*-test *p* < 0.05, *n* = 10). The effects of 4-AP on AP duration and AHP amplitude are summarized in **Figures 2C,D**, respectively.

**FIGURE 3:**
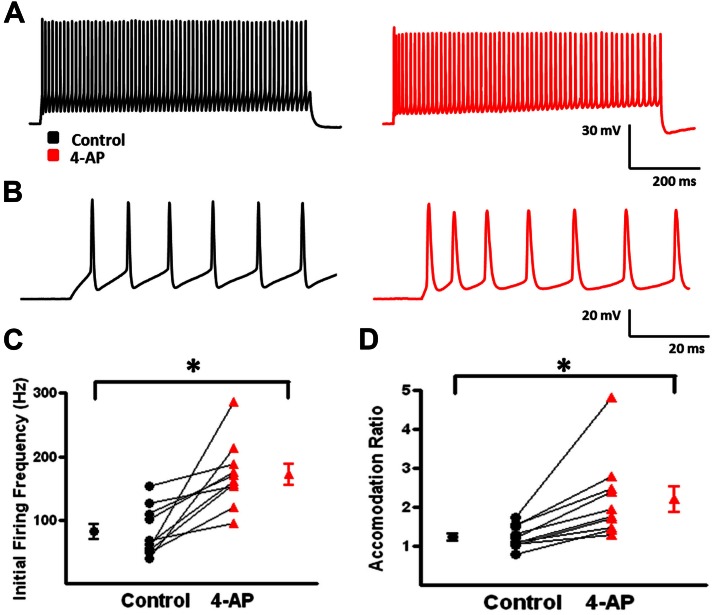
**4-aminopyridine induced changes in the repetitive firing properties of FS-BCs. (A)** Representative response of a FS-BC to a 400 pA depolarizing current pulses before (black) and after (red) bath application of 4-AP. **(B)** Expanded view of the initial firing at the onset of responses shown in **A**. 4-AP induced an increase in the initial firing frequency. **(C)** Summary plot illustrating that bath application of 4-AP significantly increases the initial firing frequency at the onset of a depolarizing current (*n* = 10). **(D)** Summary plot showing that the accommodation ratio, calculated by dividing the first interspike interval by the last interspike interval, was significantly increased after bath application of 4-AP, indicating that accommodation was increased (*n* = 10). ^∗^*p* < 0.05.

A-type K^+^ channels have been implicated in the repolarization of APs and, therefore, repetitive firing properties of certain subclasses of interneurons (Massengill et al., 1997; Erisir et al., 1999; Lau et al., 2000; Rudy and McBain, 2001). These alterations in repetitive firing properties can lead to changes in network activity (Lau et al., 2000; Harvey et al., 2012). To determine if 4-AP induced similar alterations in repetitive firing properties in neocortical FS-BCs and possibly inhibitory network activity, suprathreshold depolarizing current pulses were applied for 800 ms to measure repetitive firing properties. L2/3 FS-BCs demonstrated sustained firing throughout the current pulse with no significant accommodation (**Figures 3A,B**). Upon application of 4-AP, burst firing was induced at the onset of the depolarizing current pulse (**Figures 3A,B**). As shown in **Figure 3C**, the initial firing frequency, calculated from the first interspike interval, significantly increased in the presence of 4-AP (control: 81.7 ± 12.2 Hz, 4-AP: 172 ± 16.4; *t*-test *p* < 0.05, *n* = 10). A ratio of the first and last interspike intervals was determined before and after wash-in of 4-AP. As shown in **Figure 3D**, this ratio was near 1 under control conditions indicating little or no accommodation. The ratio was significantly decreased showing firing frequency was significantly decreased in the presence of 4-AP, indicating increased accommodation. These results suggest that inhibition of A-type K^+^ channels with 4-AP significantly alters the AP and repetitive firing properties of neocortical L2/3 FS-BCs.

#### Martinotti Cells

Martinotti cells express mRNA for all three major subclasses of A-type K^+^ channels (Wang et al., 2004), but it is unclear how these channels contribute to the intrinsic and repetitive firing properties of these critical feed-back inhibitory cells. To address this, recordings were made from L2/3 Venus-positive MCs. It is well established that MCs are a heterogeneous subclass of interneurons and can be further classified based on their repetitive firing properties (Wang et al., 2004; Xu et al., 2006; McGarry et al., 2010). In this series of experiments, two electrophysiologically distinct classes of MCs were encountered: classical accommodating (c-AC; 3/13) and burst accommodating (b-AC) MCs (10/13 cells; Wang et al., 2004). Only b-AC MCs were examined and analyzed for the following set of experiments.

**FIGURE 4:**
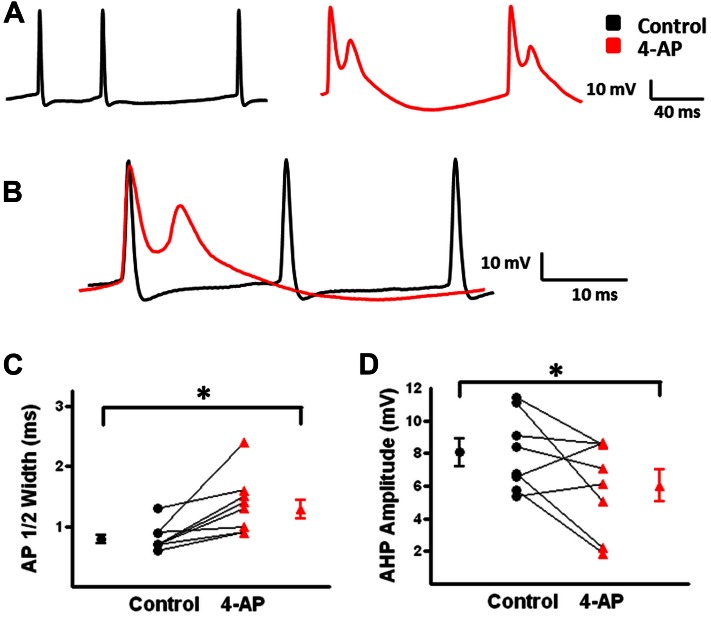
**Action potential (AP) properties of b-AC Martinotti cells (b-AC MCs) are modified in the presence of 4-AP. (A)** Representative AP firing of a L2/3 b-MC during a 475 pA depolarizing current pulse before (black) and after (red) bath application of 4-AP. **(B)** Superimposition of APs from A before and after bath application of 4-AP showing spike widening and burst firing in presence of 4-AP. **(C)** Summary plot demonstrates that bath application of 4-AP significantly increases AP duration in MCs (*n* = 10). **(D)** Summary graph illustrating that the AHP after each AP in b-AC MCs was significantly reduced by bath application of 4-AP (*n* = 10). ^∗^*p* < 0.05.

**FIGURE 5:**
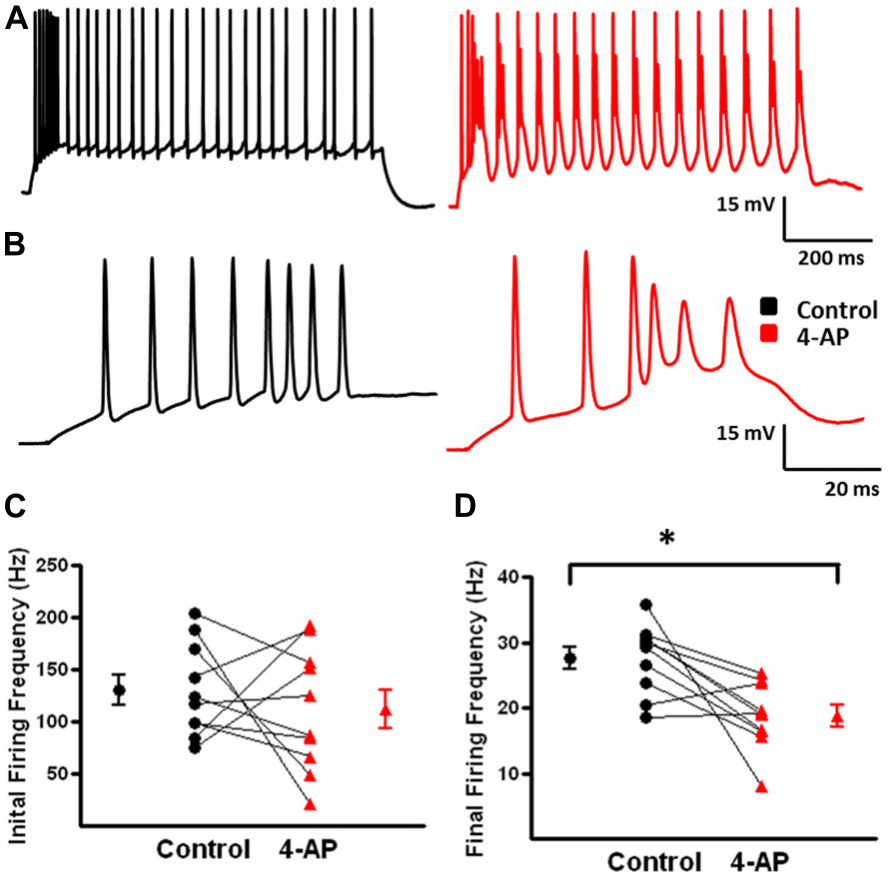
**Alterations in repetitive firing of b-AC MCs following bath application of 4-AP. (A)** Representative response of a b-AC MC to a 475 pA depolarizing current pulse before (black) and after (red) bath application of 4-AP. **(B)** An expanded view of the initial firing pattern at the onset of a 475 pA depolarizing current pulse before and after bath application of 4-AP. Enhanced, continuous, burst firing was seen in presence of 4-AP. **(C)** Summary plot illustrating that the initial firing frequency at the onset of a depolarizing current pulse is not altered by 4-AP (*n* = 10). **(D)** Summary plot showing that the final firing frequency of b-AC MCs is significantly decreased after bath application of 4-AP (*n* = 10). ^∗^*p* < 0.05.

**FIGURE 6:**
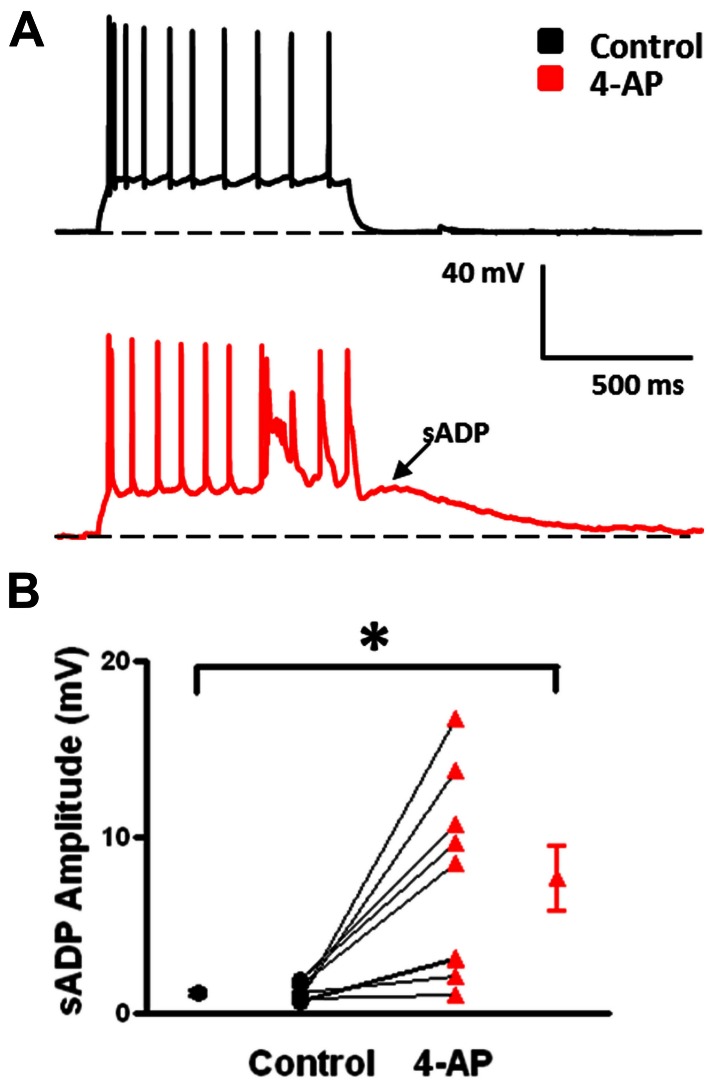
**Emergence of slow afterdepolarizations (sADPs) in b-AC MCs in presence of 4-AP. (A)** Representative response of a L2/3 b-AC MC to a 475 pA depolarizing current pulse before (black) and after (red) bath application of 4-AP. Following application of 4-AP, a sADP was observed following repetitive firing. **(B)** Summary plot showing that a significant s-ADP was observed in all b-AC MCs in presence of 4-AP (*n* = 10). *p < 0.05.

Under control conditions, b-AC MCs fired a burst of APs at onset of a depolarizing current pulse followed by a slowly accommodating train of single APs (**Figure 5A**, black). Following application of 4-AP, the same current pulse elicited repetitive burst firing (**Figure 5A**, red). Initial burst responses are shown at higher time resolution in **Figure 5B**. In the presence of 4-AP, single APs were transformed into bursts in a subset of cells; small, prolonged APs were observed following the initial AP in these burst (**Figures 4A,B**). AP half width was significantly increased by 4-AP (control: 0.8 ± 0.1 ms, 4-AP: 1.3 ± 0.2; *t*-test *p* < 0.05, *n* = 10; **Figure 4C**). The amplitude of the AHP following individual APs was also significantly decreased in the presence of 4-AP (control: 8.1 ± 0.7 mV, 4-AP: 6.2 ± 0.8 mV; *t*-test *p* > 0.05, *n* = 10; **Figure 4D**). In contrast to FS-BCs, the initial firing frequency at the onset of the depolarizing current injection did not change following 4-AP application (control: 130.4 ± 14.1 Hz, 4-AP: 112.3 ± 18.7 Hz; *t*-test *p* > 0.05, *n* = 10; **Figure 5C**). However, firing following the initial burst displayed increased accommodation with cells firing at a lower frequency (control: 27.7 ± 1.7, 4-AP: 18. 8 ± 1.6 Hz; *t*-test *p* < 0.05, *n* = 10; **Figure 5D**). In addition to the above described changes, a slow afterdepolarization (sADP) was observed in presence of 4-AP (control: 1.2 ± 0.2 mV, 4-AP: 7.6 ± 1.9 mV; *t*-test *p* < 0.05, *n* = 9; **Figure 6**). These results suggest that alterations in the firing patterns of b-AC MCs could alter network excitability and increase synchronization.

### Inhibition of I_h_ Increases Synchronous GABAergic Network Activity

GABAergic interneurons in rat neocortex do not typically stain with HCN channel antibodies (Lorincz et al., 2002; Notomi and Shigemoto, 2004). However, there is electrophysiological evidence for the presence of functional HCN channels in specific subclasses of neocortical interneurons (Lupica et al., 2001; Aponte et al., 2006; Ma et al., 2006). The role of HCN channels in excitability of GABAergic interneurons and synchronous GABAergic network activity is poorly understood. In neocortex and hippocampus, application of 4-AP, in the presence of CNQX and D-APV to block excitatory glutamatergic transmission, induces synchronized bursting of inhibitory interneurons (Avoli, 1990; Aram et al., 1991). We first examined the effects of I_h_ inhibition on inhibitory network activity in MCs, which express the largest I_h_ current among interneurons (Ma et al., 2006).

#### Martinotti Cells

**FIGURE 7:**
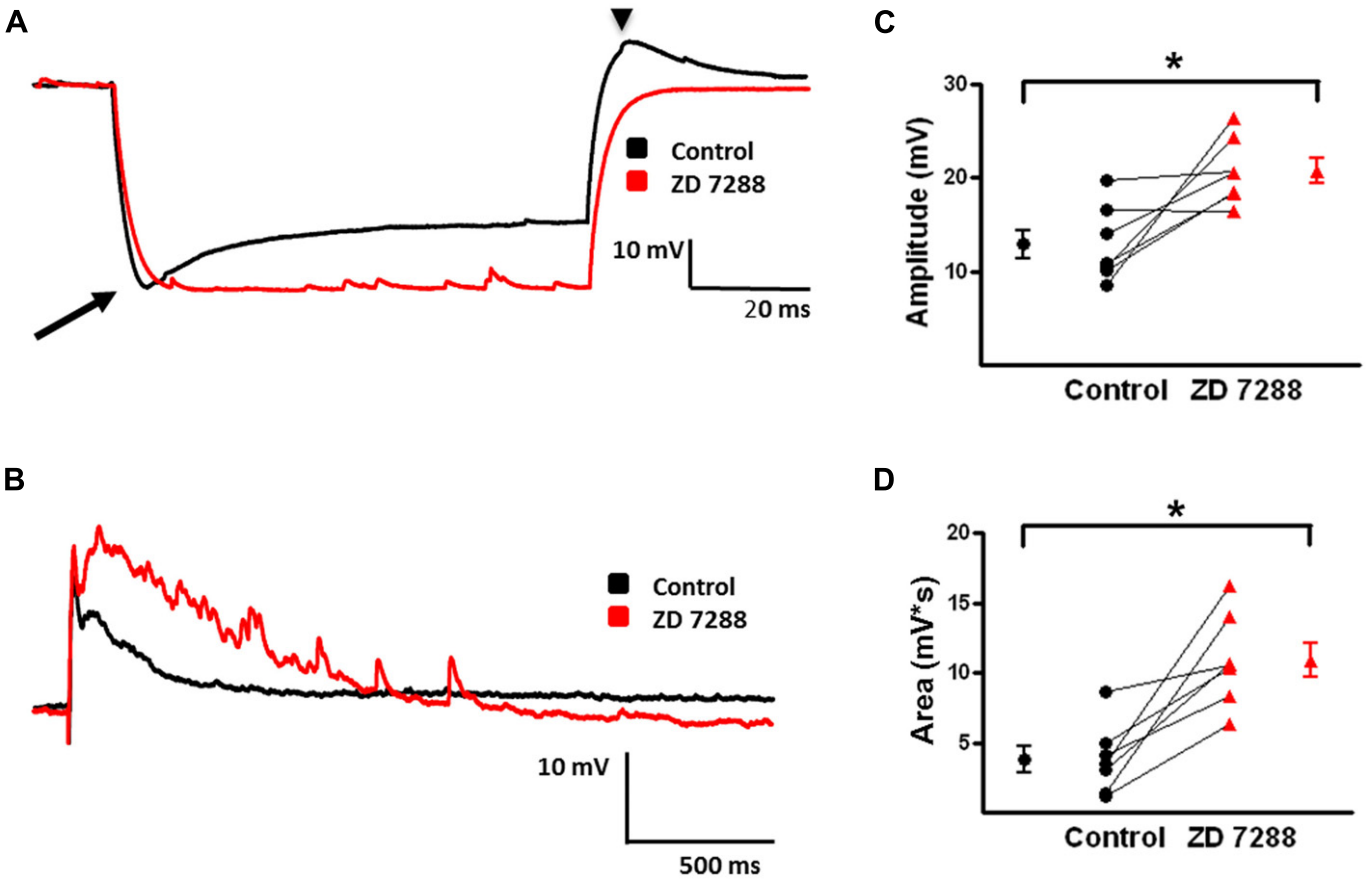
**Effect of I_h_ inhibition with ZD 7288 on magnitude of evoked depolarizing GABAergic potentials in MCs. (A)** Superimposition of representative responses of a L2/3 MC to a 250 pA hyperpolarizing current pulse. Characteristic sag (arrow) and rebound responses (arrow head) observed under control conditions (black) were inhibited following application of ZD 7288 (red). **(B)** Representative responses of evoked depolarizing GABAergic potentials in a L2/3 MC before (black) and after (red) bath application of ZD 7288. **(C)** Summary graph showing a significant increase in the amplitude of evoked depolarizing GABAergic potentials in MCs upon inhibition of I_h_. **(D)** Summary plot indicating that ZD 7288 significantly increases the area of evoked depolarizing GABAergic potential responses in MCs (*n* = 7). ^∗^*p* < 0.05.

I_h_ activation is associated with a “sag” in the peak voltage response evoked by large hyperpolarizing current pulses coupled with a rebound depolarization on pulse offset (Lupica et al., 2001; Ma et al., 2006). **Figure 7A** shows a sag (arrow) and rebound (arrow head) response in an identified MC. ZD 7288, a specific HCN channel antagonist, reduced both sag and rebound responses when bath applied at 20 μM (**Figure 7A**). In the presence of 4-AP, CNQX, and D-APV, electrical stimulation in L2/3 reliably produced a depolarizing GABAergic response in the recorded MCs. With the given intracellular Cl^-^ concentration of the pipette solution and the extracellular Cl^-^ concentration of the bath solution, the calculated reversal potential for evoked GABAergic responses is -66 mV. These responses are inhibited by bicuculline (20 uM), indicating they are GABAergic in nature (data not shown; see also Aram et al., 1991). Inhibition of HCN channels with ZD 7288 enhanced evoked depolarizing GABAergic responses (**Figure 7B**). Summary graphs in **Figures 7C,D** show that ZD 7288 significantly increased evoked depolarizing GABAergic response amplitude and area, respectively. These results suggest that HCN channels, presumably via I_h_, normally restrict network activity in this model of GABAergic synchronization.

**FIGURE 8:**
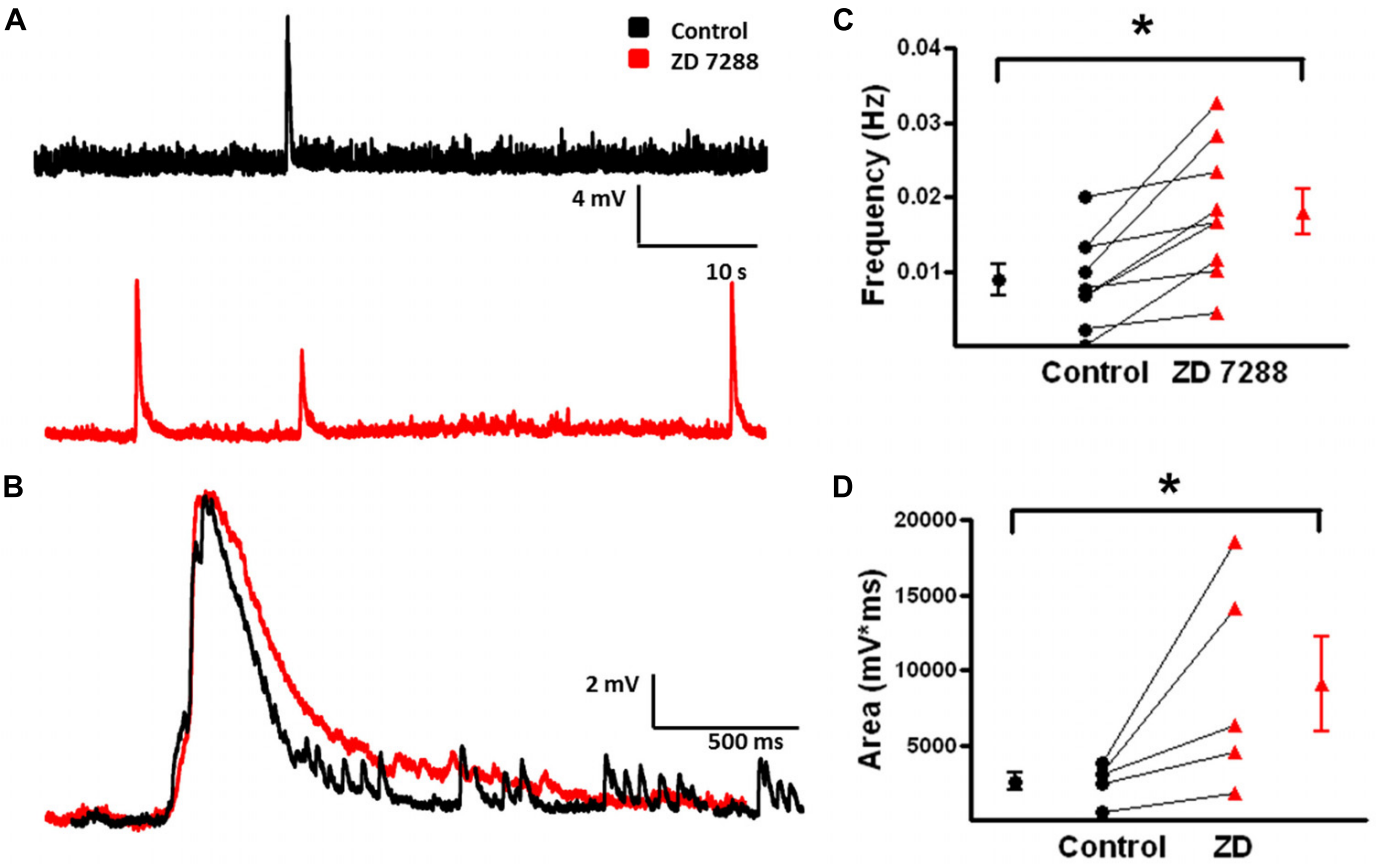
**Frequency and area of spontaneous depolarizing GABAergic potentials in L2/3 MCs increase with inhibition of I_h_. (A)** Specimen records showing spontaneous depolarizing GABAergic potentials before (black) and after (red) bath application of ZD 7288. A significant increase in the frequency of depolarizing GABAergic potentials was observed following inhibition of I_h_. **(B)** An expanded view of a single spontaneous depolarizing GABAergic potential before (black) and after (red) bath application of ZD 7288. **(C)** Summary plot demonstrating a significant increase in the frequency of spontaneous GABAergic events following application of ZD 7288 (*n* = 9). **(D)** Summary graph demonstrating that inhibition of I_h_ significantly increases the area of spontaneous depolarizing GABAergic potentials. (*n* = 5). ^∗^*p* < 0.05.

We further investigated the effect of I_h_ inhibition on network activity by measuring spontaneously occurring depolarizing GABAergic responses in L2/3 MCs. Examples of spontaneous depolarizing GABAergic responses are shown in **Figures 8A,B**. In the presence of 4-AP, CNQX, and D-APV, spontaneous events occurred at a low rate. When examined at higher time resolution, spontaneous events in MCs were seen to consist of a membrane depolarization with few or no superimposed APs (**Figure 8B**). ZD 7288 significantly increased the frequency of spontaneous depolarizing GABAergic responses (control: 0.009 ± 0.002 Hz, ZD: 0.018 ± 0.003 Hz; *t*-test *p* < 0.05, *n* = 9; **Figure 8C**). Similar to the effects on evoked depolarizing GABAergic responses, bath application of ZD 7288 significantly increased the area of spontaneous depolarizing GABAergic responses (control: 2585 ± 556 mv^∗^ms, ZD: 9079 ± 3126 mV^∗^ms; *t*-test *p* < 0.05, *n* = 5; **Figure 8D**). These results show that inhibition of HCN channels leads to an increase in the amplitude and area of evoked and spontaneously occurring depolarizing GABAergic responses, as well as an increase the frequency of spontaneous depolarizing GABAergic responses in L2/3 MCs.

#### Fast-Spiking Basket Cells

**FIGURE 9:**
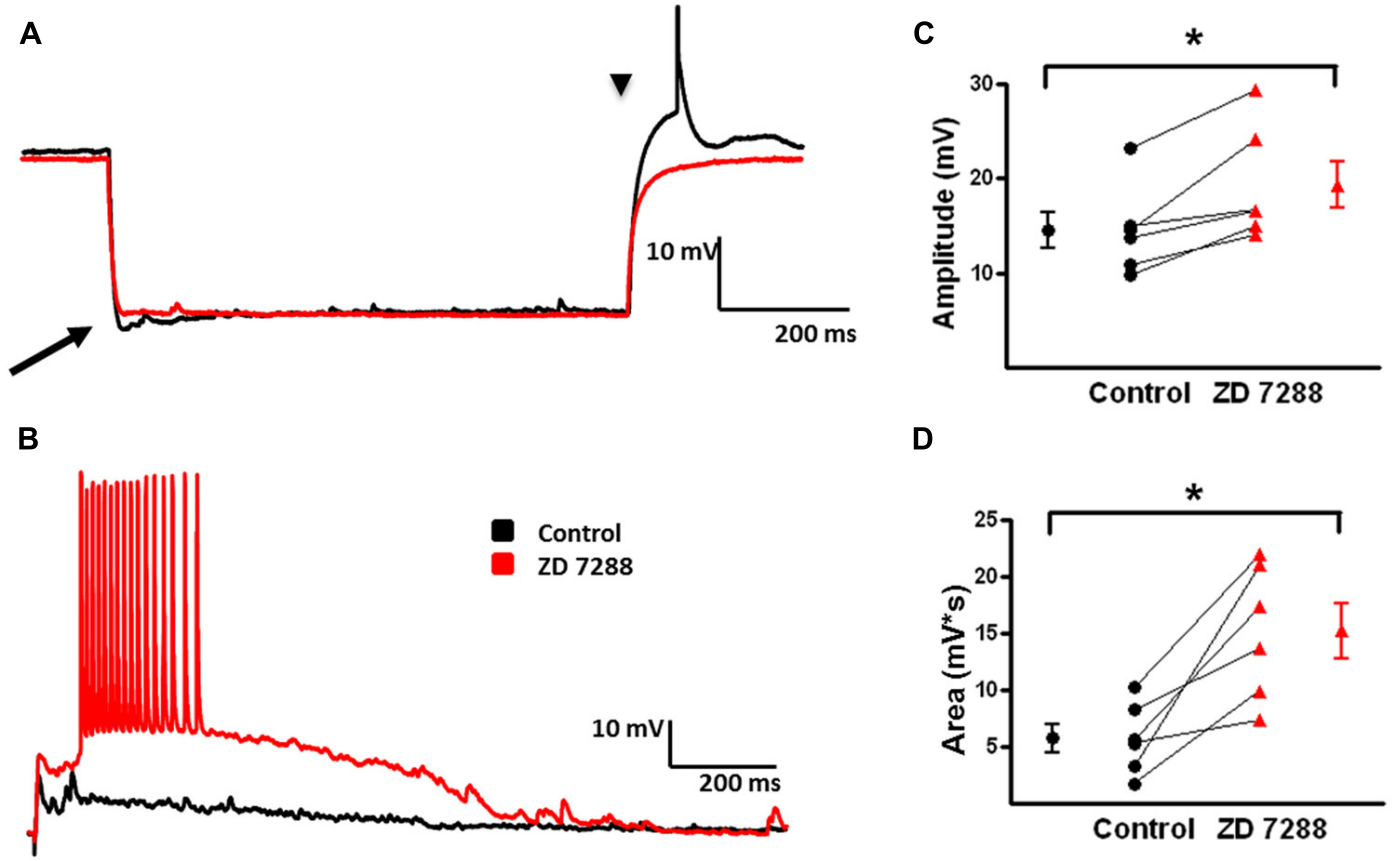
**Evoked depolarizing GABAergic potentials are enhanced in FS-BCs following I_h_ inhibition. (A)** Superimposition of representative responses of a L2/3 FS-BC to a 250 pA hyperpolarizing current pulse. Characteristic small amplitude sag (arrow) responses and rebound depolarizations (arrow head) were observed in FS-BCs under control conditions (black). Sag and rebound responses were blocked in presence of ZD 7288 (red). **(B)** Superimposed specimen records of evoked depolarizing GABAergic potentials in a L2/3 FS-BC before (black) and after (red) bath application of ZD 7288. **(C)** Summary plot demonstrating a significant increase in the amplitude of evoked depolarizing GABAergic potentials in FS-BCs upon I_h_ inhibition (*n* = 6). **(D)** Summary plot demonstrating ZD 7288 significantly increases the area of evoked depolarizing GABAergic potential responses in FS-BCs (*n* = 6). ^∗^*p* < 0.05.

These cells display small I_h_ current upon hyperpolarization (Aponte et al., 2006). Nonetheless, it was hypothesized that HCN channel inhibition would also enhance activity in FS-BCs due to enhanced network activity. Recordings were obtained from YFP-positive cells that displayed FS-BC intrinsic and repetitive firing properties, as described above. **Figure 9A** shows that hyperpolarizing current pulses were associated with small rapid sag responses (arrow) and rebound response (arrow head). ZD 7288 (20 μM) application abolished both sag and rebound responses (**Figure 9A**).

Slices were incubated in 4-AP for >1 h to develop robust synchronous network activity. In the presence of 4-AP, CNQX, and D-APV, electrical stimulation evoked depolarizing GABA-mediated responses when cells were held at -80 mV in current clamp (**Figure 9**, black). Depolarizing GABAergic responses were reliably evoked in FS-BCs (**Figure 9B**) with an average amplitude of 14.5 ± 1.9 mV and area under the curve of 5783 ± 1270 mV^∗^ms (**Figures 9C,D**). Bath application of ZD 7288 significantly increased the amplitude (ZD: 19.3 ± 2.5 mV; *t*-test *p* < 0.05, *n* = 6; **Figure 9C**) and area (ZD: 15269. ± 2428 mV*ms; *t*-test, *p* < 0.05, *n* = 7; **Figure 9D**) of evoked depolarizing GABAergic responses. In 6/7 cells, the previously subthreshold stimulation initiated large amplitude events with superimposed APs.

**FIGURE 10:**
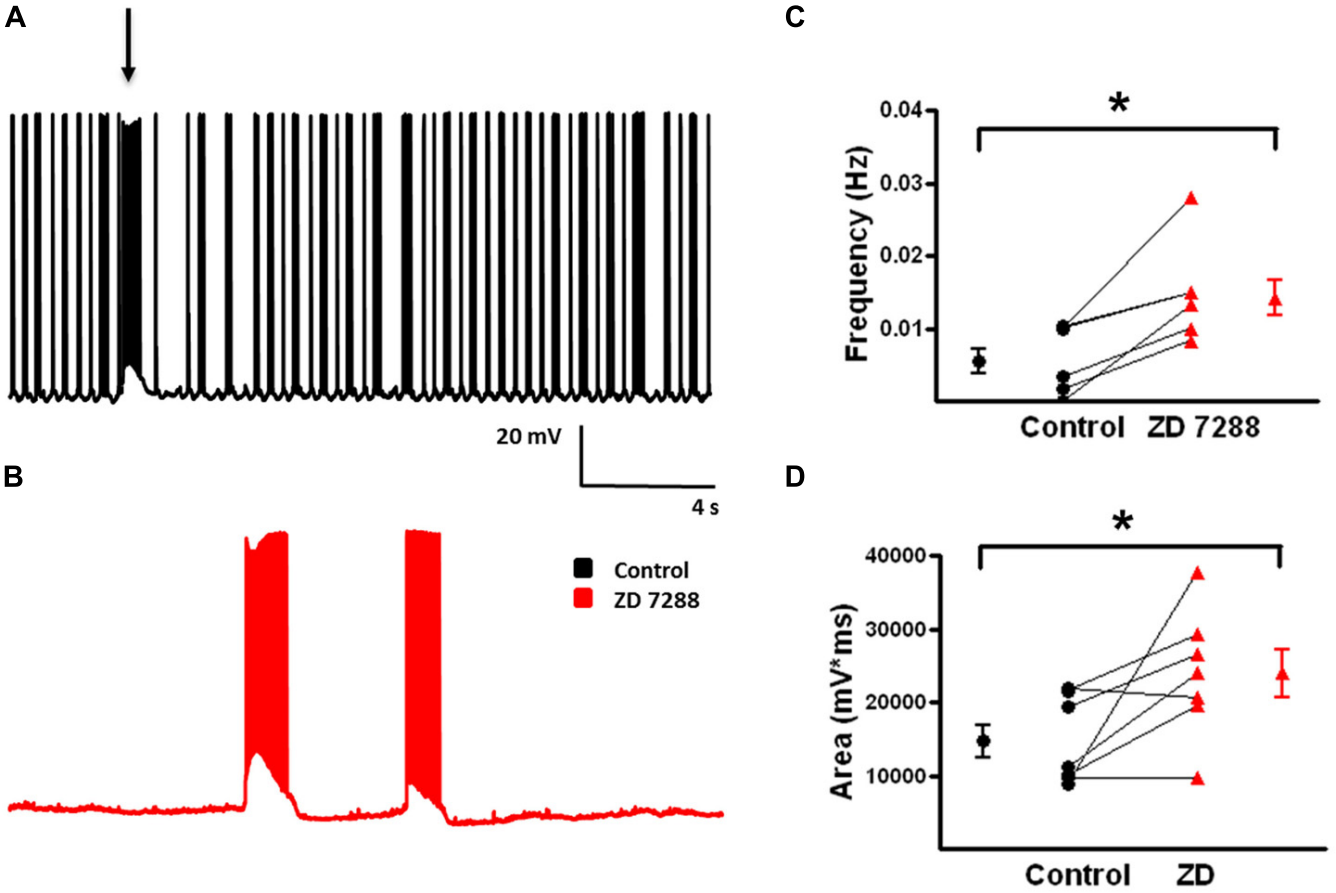
**Frequency and area of spontaneous depolarizing GABAergic potentials in L2/3 FS-BCs increase with I_h_ inhibition. (A)** Representative trace of a spontaneous depolarizing GABAergic potential (arrow) in a L2/3 FS-BC. Specimen record also shows characteristic 4-AP induced baseline burst firing in FS-BC, which precede and follow spontaneous depolarizing GABAergic potentials. **(B)** Representative trace of spontaneous events following bath application of ZD 7288. **(C)** Summary plot demonstrates that inhibition of I_h_ with ZD 7288 significantly increases the frequency of spontaneous depolarizing GABAergic potentials in FS-BCs (*n* = 7). **(D)** Summary plot demonstrates that the area of spontaneous depolarizing GABAergic potentials significantly increases with inhibition of I_h_ (*n* = 7). ^∗^*p* < 0.05.

Spontaneous depolarizing GABAergic responses with superimposed APs were observed in FS-BCs (**Figure 10A**, arrow). In addition, FS-BCs demonstrated a persistent membrane potential oscillation often accompanied by APs on the depolarizing phase (**Figure 10A**). These baseline oscillations and bursts of APs were not accompanied by a significant membrane depolarization and were not included in the analysis of depolarizing GABAergic responses. This activity was not observed following application of ZD 7288 whereas spontaneous depolarizing GABA responses were significantly enhanced (**Figure 10B**). ZD 7288 significantly increased the frequency of spontaneous depolarizing GABAergic responses in FS-BCs (control: 0.007 ± 0.002 Hz, ZD: 0.014 ± 0.003 Hz; *t*-test, *p* < 0.05, *n* = 7; **Figure 10C**). HCN channel inhibition also significantly increased the area of spontaneous depolarizing GABAergic responses (control: 14709 ± 2257 mV^∗^ms, ZD: 23951 ± 3306 mV^∗^ms; *t*-test *p* < 0.05, *n* = 7; **Figure 10D**). This increase in depolarizing GABAergic response frequency and area was accompanied by a decrease or loss of baseline oscillations observed under control conditions.

### Temporal IPSP Summation in GABAergic Interneurons

**FIGURE 11:**
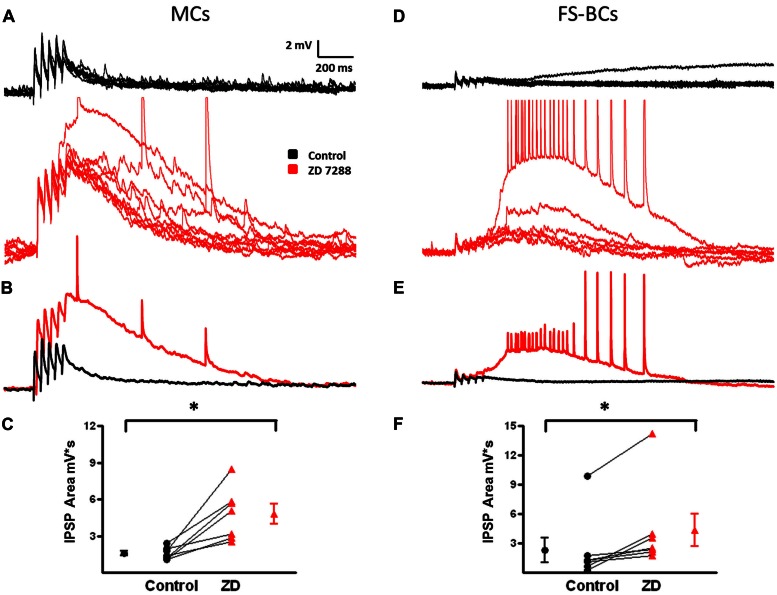
**Changes in temporal integration of depolarizing IPSP in MCs and FS-BCs after I_h_ inhibition. (A)** Ten superimposed responses of a L2/3 MC to a train of five stimuli at 25 Hz (20–50 μA) before (top, black) and after (bottom, red) bath application of Z7288. Temporal integration of IPSPs was observed under control conditions. This was significantly enhanced upon inhibition of I_h_. **(B)** Superimposed averaged responses of the individual responses shown in **(A)**. **(C)** Summary plot demonstrating ZD 7288 significantly increases in IPSP area in L2/3 MCs (*n* = 7). **(D)** Superimposed individual responses of a L2/3 FS-BC to a similar 25 Hz train before (top, black) and after (bottom, red) bath application of ZD7288. Characteristic IPSP depression was observed in the control condition. **(E)** Averaged responses of the individual traces shown in **(D)**. Alterations in IPSP depression was not observed in FS-BCs following inhibition of I_h_. However, ZD 7288 did cause the appearance of a late depolarization that could initiate depolarizing GABAergic potentials. **(F)** Summary plot illustrating that bath application of ZD 7288 significantly increases the area of depolarization after 25 Hz stimulation (*n* = 7). ^∗^*p* < 0.05.

In neocortical pyramidal cells, temporal integration of EPSPs is reduced or prevented by I_h_ and I_h_ inhibition results in enhanced synaptic integration (Berger et al., 2001). Modulation of synaptic integration in interneurons is relatively unexplored. However, it has been observed that facilitating EPSPs from pyramidal cells onto MCs were only slightly changed in the presence of ZD 7288 to block I_h_ (Berger et al., 2010). In the present study, IPSPs are depolarizing due to alterations in the Cl^-^ equilibrium potential (Aram et al., 1991). It was hypothesized that the increased network excitability observed in the presence of the HCN channel inhibitor ZD 7288 was due to changes in temporal integration. To test this, five IPSPs were evoked at 25 Hz in both MCs and FS-BCs. EPSPs in MCs typically show temporal integration (Berger et al., 2010). In MCs, when depolarizing IPSPs were evoked at 25 Hz, modest temporal integration was observed (**Figure 11A**, control). These IPSPs were associated with a pronounced underlying depolarization. Inhibition of I_h_ significantly increased the amplitude of IPSPs 1-5 (Two-way ANOVA, *p* < 0.05; **Figure 11B**). Paired pulse ratios (PPRs) for IPSPs1 and 5 in MCs were significantly increased in the presence of ZD 7288 (1.67 ± 0.3 in Control and 2.4 ± 0.3, *t*-test, *p* < 0.05, *n* = 6). The area of the underlying depolarization was significantly enhanced (**Figure 11C**) and was occasionally associated with the onset of a depolarizing GABAergic response (Control: 1566 ± 189 mV^∗^s, ZD: 4806 ± 803 mV^∗^ s; *t*-test, *p* = 0.05).

I_h_ currents and the effect of I_h_ on synaptic integration have not been extensively examined in FS-BCs. The small sag responses observed here (**Figure 9A**) led us to hypothesis that synaptic integration would not be affected in FS-BCs, but an enhanced underlying depolarization would be seen following I_h_ inhibition. Superimposed records in **Figure 11D** show that IPSPs displayed depression during the train of 25 Hz stimulation. Averages of these responses are shown in **Figure 11E**. Inhibition of I_h_ had no significant effect on the amplitude of first IPSP (Control: 3.3 ± 0.4 mV, ZD: 3.2 ± 0.4 mV; *t*-test, *p* = 0.25) or IPSPs 1–5 (two-way ANOVA, *p* = 0.08). PPRs were not significantly changed in presence of ZD 7288 (0.575 ± 0.1 Control and 0.86 ± 0.1 in presence of ZD 7288; *t*-test, *p* < 0.05, *n* = 4). I_h_ inhibition was, however, accompanied by a increased late depolarization and associated with evoked depolarizing GABAergic responses (Control: 2314 ± 1276 mV^∗^ s, ZD: 4354 ± 1671 mV^∗^ s; *t*-test, *p* = 0.05; **Figure 11F**). These results suggest that HCN channels differentially modulate synaptic integration of depolarizing IPSPs in MCs compared to FS-BCs.

## Discussion

This study investigated the effect of 4-AP on the firing properties of L2/3 FS-BCs and MCs. In the presence of 4-AP, AP durations increased and AHP amplitudes decreased in both L2/3 FS-BCs and MCs. FS-BCs demonstrated an increase in initial firing frequency upon suprathreshold depolarization and an increase in accommodation in the presence of 4-AP. Initial burst firing was maintained in b-AC MCs and persistent repetitive burst firing was also observed. Additionally, we investigated the effect of HCN channel inhibition on GABAergic network synchronization. In the presence of ZD 7288 to inhibit I_h_, the area of evoked GABAergic depolarizations was increased in both FS-BCs and b-AC MCs. Similarly, inhibition of I_h_ increased the area and frequency of spontaneous depolarizing GABAergic events in these cells. These results indicate that 4-AP differentially alters the intrinsic firing patterns of interneurons. Acute inhibition of HCN channels enhances GABAergic interneuron synchronization indicating that I_h_ modulates inhibitory network excitability.

### 4-AP Alters the Intrinsic Firing Properties of FS-BCs and b-MCs

The mechanism by which application of 4-AP in the presence of CNQX and D-APV induces GABA_A_ receptor-mediated synchronization of neocortical GABAergic interneurons remains unclear. Early studies with 4-AP examined role of potassium channels in synaptic transmission at the squid giant synapse (Llinas et al., 1976). It was later shown to have convulsant effects *in vitro* and *in vivo* (Spyker et al., 1980; Thesleff, 1980; Jack et al., 1981; Voskuyl and Albus, 1985; Rutecki et al., 1987; Szente and Baranyi, 1987; Mihaly et al., 1990; Perreault and Avoli, 1991). Subsequent investigation into the mechanism of action concluded that 4-AP blocks transient outward K^+^ currents through A-type K^+^ channels in a variety of systems (Hermann and Gorman, 1981; Choquet and Korn, 1992; Mei et al., 1995). Additionally, 4-AP blocks the delayed K^+^ current I_D_ (Storm, 1988; Wu and Barish, 1992; Barish et al., 1996; Coetzee et al., 1999).

Application of 4-AP enhances release of both excitatory and inhibitory neurotransmitters (Rutecki et al., 1987; Tibbs et al., 1989; Perreault and Avoli, 1991; Otis and Mody, 1992). A-type K^+^ channels are predominantly expressed in the soma and dendrites of pyramidal neurons, increasing in density distal to the soma, with sparse expression in axons and terminals (Hoffman et al., 1997; Bekkers, 2000; Korngreen and Sakmann, 2000; Johnston et al., 2003; Dodson and Forsythe, 2004; Foust et al., 2011). These channels, mainly consisting of Kv4 subunits, in addition to shaping the AP waveform, regulate back propagation of APs into distal dendrites, and control excitability and output of pyramidal neurons (Stuart et al., 1997; Bekkers, 2000; Kang et al., 2000; Yuan et al., 2005). The isoform composition and cellular localization of A-type K^+^ channels is more variable in interneurons compared to pyramidal cells (Weiser et al., 1995; Du et al., 1996; Martina et al., 1998; Chow et al., 1999; Coetzee et al., 1999).

The dissimilar effects of 4-AP on AP and repetitive firing properties in FS-BCs and MCs observed here may be attributable to differential expression and localization of A-type K^+^ channels in various classes of interneurons (Chow et al., 1999; Erisir et al., 1999; Jerng et al., 2004; Goldberg et al., 2008; Rudy et al., 2011). In the presence of 4-AP, we observed that AP duration was similarly increased in both FS-BCs and MCs, consistent with a known global enhancement of synaptic transmission (Jankowska et al., 1977; Buckle and Haas, 1982; Otis and Mody, 1992). The characteristic fAHP of FS-BCs was markedly reduced and accommodation was increased. Axons and nerve terminals of FS-BCs express Kv3 and Kv1 which may directly affect the AP repolarization in these structures (Massengill et al., 1997; Erisir et al., 1999; Lau et al., 2000; Goldberg et al., 2008). The blockade of the Kv1 current is most likely responsible for the switch from a single AP to a burst of APs at the onset of a depolarizing current pulse (Wu and Barish, 1992; Goldberg et al., 2008). Furthermore, Kv3 channels are necessary for the FS phenotype of FS-BCs (Rudy and McBain, 2001; Lien and Jonas, 2003). The inhibition of Kv3 channels likely resulted in the observed increase in AP accommodation in the present study.

In contrast to FS-BCs, MCs express the Kv4 family of channels at a higher density in the somatodendritic region. It remains unknown if these channels are expressed in a similar gradient pattern along the dendrites in interneurons as seen in pyramidal neurons. However, they function to tightly regulate dendritic Ca^2+^ spikes and back-propagating APs in other cells (Serodio and Rudy, 1998; Lien et al., 2002; Chen and Johnston, 2004; Lai and Jan, 2006; Bourdeau et al., 2007; Sun et al., 2011). Our observed effects of 4-AP on repetitive firing properties in neocortical MCs can be attributed to blockade of Kv4 channels. By blocking Kv4 channels, back-propagating APs and Ca^2+^ spikes would be able to antidromically invade the dendrites causing a prolonged depolarization and possibly initiating further AP firing, directly contributing the transformation of single APs to repetitive burst responses observed in MCs. Additionally, the dendritic hyperexcitability resulting from inhibition of somatodendritic Kv4 channels could underlie the unmasking of a sADP following repetitive firing in the presence of 4-AP.

### 4-AP Induced GABAergic Network Synchronization

GABAergic synapse formation often precedes that of excitatory glutamatergic synapses (Ben-Ari et al., 1989). Since immature neurons have higher intracellular chloride concentrations due to early lack of KCC2 chloride transporters, GABA depolarizes and excites immature neurons (Ben-Ari et al., 2007). Early in development, excitatory GABAergic transmission is associated with spontaneous giant depolarizing potentials (Garaschuk et al., 1998; Bonifazi et al., 2009). Similar appearing responses are seen in more mature animals under appropriate pharmacological conditions. In the presence of glutamatergic antagonists, bath application of 4-AP induces a GABA_A_-receptor mediated network synchronization of interneurons (Aram et al., 1991; Avoli et al., 1998). This hyperexcitability in inhibitory networks produces large amplitude depolarizing potentials that propagate through the cortex (DeFazio and Hablitz, 2005). The enhancement in inhibitory synaptic transmission associated with broadened APs could produce an increase in the intracellular Cl^-^ concentration through the activation of GABA-gated chloride channels producing an activity-dependent shift in the concentration gradient for Cl^-^ resulting in the observed membrane depolarization (Ling and Benardo, 1995; Staley et al., 1995). The synchronous firing of neurons associated with depolarizing GABA responses produces an elevation in the extracellular K^+^ concentration contributing to the persistence of interneuron synchronization (Gilbert et al., 1984; MacVicar et al., 1989; Avoli et al., 2002). Synchronous GABAergic discharges have been predominantly investigated in brain slice preparations. However, co-perfusion of 4AP and glutamate receptor blockers in an *in vitro* isolated guinea pig brain preparation produced spontaneous synchronized propagating events that exhibited sensitivity to the GABA_A_ receptor antagonist bicuculline (Uva et al., 2009). It is likely that synchronization of GABAergic networks also occurs *in vivo*. The intrinsic changes that occur in interneurons to initiate this form of synchronous activity are not well understood. The subclasses of interneurons that initiate and participate in network synchronization have not been clearly established.

We observed robust spontaneous and evoked GABAergic depolarizations in FS-BCs and MCs in the presence of 4-AP and glutamatergic antagonists. The GABAergic depolarizations observed in FS-BCs were noticeably different from those seen in MCs. Large GABAergic depolarizations in FS-BCs were accompanied by high frequency firing. Similar firing has been seen in other fast spiking interneurons (Benardo, 1997; Keros and Hablitz, 2005). GABAergic depolarizations rarely initiated AP firing in MCs. Differential expression and localization of 4-AP sensitive channels in FS-BCs versus MCs may contribute to this variability. The inhibition of Kv1 and Kv3 currents in the axons of FS-BCs can lower threshold and increase the onset of AP firing (Rudy and McBain, 2001; Goldberg et al., 2008). The repetitive firing of FS-BCs in association with GABAergic depolarizations in the presence of 4-AP may contribute to network synchronization and support propagation of synchronous activity.

In addition to firing APs in coordination with GABAergic depolarizations, FS-BCs repeatedly fired single APs or bursts of APs from baseline. These ectopic APs (EAPs) are generated distally in either the axons or dendrites and propagate into the soma (Gutnick and Prince, 1974; Stasheff et al., 1993). EAPs are known to be associated with 4-AP induced spontaneous GABAergic depolarizations (Perreault and Avoli, 1989; Avoli et al., 1998) and have been observed previously in GABAergic interneurons (Benardo, 1997; Keros and Hablitz, 2005). FS-BC are known to rapidly synchronize due to the high incidence of reciprocal connections and gap junctions, allowing them to initiate cortical gamma oscillations and control theta oscillations in the hippocampus (Pouille and Scanziani, 2001; Connors and Long, 2004; Cardin et al., 2009; Pouille et al., 2009). The enhanced output of FS-BCs associated with GABAergic depolarizations in combination with their electrical coupling through gap junctions suggests a role in the initiation and propagation of the depolarizing GABA responses observed here.

Fast-spiking basket cells and MCs are only two classes of interneurons out of many in the neocortex. A third interneuron subclass of great interest in the regulation of network synchronization is the neurogliaform (NGF) cell. Unlike many other interneuron subclasses, NGFs form electrical synapses with homologous and heterologous interneurons (Zsiros and Maccaferri, 2005), making them critical in the generation of synchronized neuronal network activity (Price et al., 2005). Also, this subclass of interneuron can uniquely modulate local network activity through volume transmission or non-synaptic transmission (Olah et al., 2009). Further investigation of the effects of 4-AP on the firing properties of NGFs and of 4-AP induced synchronous events in these cells would provide insight into their role in modulating GABAergic network synchronization.

### I_h_ Modulation of Network GABAergic Synchronization

The inhibitory role of I_h_ in regulating the excitability of pyramidal neurons is well established (Magee, 1999; Williams and Stuart, 2000; Berger et al., 2001). I_h_ also acts to constrain epileptiform activity in the L5 pyramidal cells (Albertson et al., 2013). Genetic deletion of HCN1 channels in the forebrain enhances theta oscillations in the hippocampus (Nolan et al., 2004). Additionally, loss of HCN channels enhances oscillatory activity related to epileptic activity in the neocortex (Strauss et al., 2004; Kole et al., 2007). How HCN channels modulate synaptic integration in GABAergic interneurons and synchronous neocortical inhibitory network activity remains unclear.

Hyperpolarization-activated cyclic nucleotide-gated channels and I_h_ have been shown to be present in both FS-BCs and MCs at varying densities (Kilb and Luhmann, 2000; Santoro et al., 2000; Wang et al., 2004; Luján et al., 2005; Wu and Hablitz, 2005; Aponte et al., 2006; Albertson et al., 2013). In the present study, MCs displayed robust I_h_ activation upon hyperpolarization whereas minimal I_h_ responses were observed in FS-BCs. Despite the difference in I_h_ expression between FS-BCs and MCs, a significant enhancement of the amplitude and area of evoked and spontaneous synchronous GABAergic events was observed in both cell types after inhibition of HCN channels. Furthermore, the frequency of spontaneous events was also enhanced in both types of interneurons. The increase in amplitude and area suggests an effect on the presynaptic release of GABA possibly caused by a depolarization of the terminals after I_h_ inhibition, consistent with previous findings (Southan et al., 2000; Aponte et al., 2006; Boyes et al., 2007). The increase in frequency may be attributable to enhanced intrinsic excitability, perhaps resulting in recruitment of small synchronized groups of interneurons to a point where a propagating network event occurs. I_h_ inhibition was associated with a loss or decrease in spontaneous EAP firing between synchronous depolarizing GABAergic events. In rodent pain models, HCN channel inhibition has been shown to decrease spontaneous APs and burst firing in dorsal root ganglion cells (Chaplan et al., 2003; Lee et al., 2005; Sun et al., 2005).

We further investigated the mechanism by which I_h_ inhibition modulated network activity by examining changes in synaptic integration. Excitatory inputs in MCs show facilitation whereas FS-BCs show depression (Buhl et al., 1997; Galarreta and Hestrin, 1998; Reyes et al., 1998; Gil et al., 1999; Wang et al., 2004; Silberberg and Markram, 2007; Thomson and Lamy, 2007). Similar findings were observed here with depolarizing IPSPs. Depolarizing IPSPs evoked at 25 Hz in MCs were facilitating. Facilitation and the underlying depolarization were enhanced by inhibition of I_h_ with ZD 7288. In FS-BCs, synaptic responses showed depression. Following inhibition of I_h_, no significant effect on synaptic depression was observed in FS-BCs. However, a delayed synchronous GABAergic network event was reliably evoked. This suggests that inhibition of I_h_ was associated with significant excitability changes in other neurons which resulted in a propagating response that was seen in FS-BCs.

The subcellular distribution of HCN channels in cortical MCs and FS-BCs may be the cause for the differences we observed in IPSP summation in these two cell types. Given that MCs exhibit large I_h_ currents upon hyperpolarization, it is assumed that there is a corresponding high density of HCN channels. Since ZD 7288 caused an increase in summation of depolarizing IPSPs, it is possible that HCN channels are located in the somatodendritic region of MCs where they would be able to exert control over synaptic regulation. We also observed an increase in IPSP area with summation in MCs, consistent with somatodendritic localization of HCN channels. Conversely, presynaptic localization of HCN channels in cortical FS-BC may account for the small density of I_h_ recorded in the soma and lack of an effect on somatodendritic summation of synaptic inputs. Functional HCN channels have been localized in the axon and presynaptic terminal of interneurons in the hippocampus, cerebellum, and basal ganglia and have been shown to regulate GABAergic synaptic transmission (Southan et al., 2000; Aponte et al., 2006; Boyes et al., 2007). Inhibition of I_h_ with ZD 7288 results in an increase in miniature inhibitory postsynaptic currents (mIPSCs), suggesting a presynaptic mechanism of action (Southan et al., 2000; Boyes et al., 2007). It is possible that the inhibition of HCN channels in the synaptic terminal causes hyperexcitability of the terminal resulting in an increase of GABA release, thus leading to a further enhancement of GABAergic network synchronization. This is consistent with our results showing that the robust firing associated with depolarizing GABAergic events in FS-BCs is significantly enhanced following the inhibition of I_h_. Further studies are needed to directly determine if release probability is regulated by HCN channels.

In summary, the data presented here show that 4-AP differentially alters the AP and repetitive firing properties of FS-BC and MCs in the neocortex. MCs and FS-BCs display different patterns of activity during depolarizing GABA responses suggesting different classes of interneurons subserve diverse roles in generation and propagation of these responses. Although the role of HCN channels in regulating normal GABAergic synaptic transmission needs to be established, our results suggest that HCN channels restrict inhibitory synaptic transmission as well as network activity in the presence of 4-AP, CNQX, and D-APV.

## Conflict of Interest Statement

The authors declare that the research was conducted in the absence of any commercial or financial relationships that could be construed as a potential conflict of interest.
